# Supplementary material for: Effective End-Group Modification of Star-Shaped PNVCL from Xanthate to Trithiocarbonate Avoiding Chemical Crosslinking
Source: Polymers (Basel). 2021 Oct 25;13(21):3677. doi: 10.3390/polym13213677 (PMC8588085; doi:10.3390/polym13213677)
Supplement: Supplementary file 1 [file polymers-13-03677-s001.zip › polymers-1430927-supplementary.pdf]

polymers

MDPI

Article

Effective End-Group Modification of Star-Shaped PNVCL from Xanthate to Trithiocarbonate Avoiding Chemical Crosslinking

Norma A. Cortez-Lemus *, Eduardo Hermosillo-Ochoa and Ángel Licea-Claverie

Centro de Graduados e Investigación en Química, Tecnológico Nacional de México/Instituto Tecnológico de Tijuana, A. P. 1166., Tijuana 22000, Mexico; eduardo.hermosillo17@tectijuana.edu.mx (E.H.-O.); aliceac@tectijuana.mx (Á.L.-C.)

* Correspondence: ncortez@tectijuana.mx

Table S1. Characteristics of PNVCL star polymers prepared in this work.

Entry	Sample[a]	$[NVCL]_o$: $[CTA]_o$	Time (h)	Conv.[b] (%)	$M_{n\ theo}$[c] (g/mol)	$M_{n\ GPC}$[d] (g/mol)	$Đ$[d]	LCST[e]	$(PNVCL\text{-}SH)_6$ $M_{n\ GPC}$[d] (g/mol)	$Đ$[d]
1	$(PNVCL_{48})_6$	430	24	64	39,565	39,610	1.15	-	47,260	1.07
2	$(PNVCL_{32})_6$	430	18	-	-	26,900	1.13	30	27,500	1.2
3	$(PNVCL_{55})_6$	480	18	55	38,000	46,100	1.11	36	-	-
4	$(PNVCL_{35})_6$	430	20	54	34,188	29,200	1.24	28	43,560	1.22
5	$(PNVCL_{25})_6$	430	16	42	26,415	21,100	1.09	24	-	-

The polymerization reaction was carried out in *p*-dioxane at 30 °C in the presence of hexafunctional xanthate-type. [a]The subscript numbers represent the repeating units of PNVCL estimated by using GPC. [b]Determined gravimetrically. [c]Calculated using equation: $M_{n\ theo} = M_{CTA}$ + conv. $(M_{NVCL}[NVCL]_o/[CTA]_o)$. [d]Determined by GPC in THF at 35 °C with RI and LS detectors using polystyrene linear standards for calibration of LS detector. PNVCL *dn/dc* = 0.109 mL/g. [e]Determined by DLS in aqueous water (1 mg/mL).

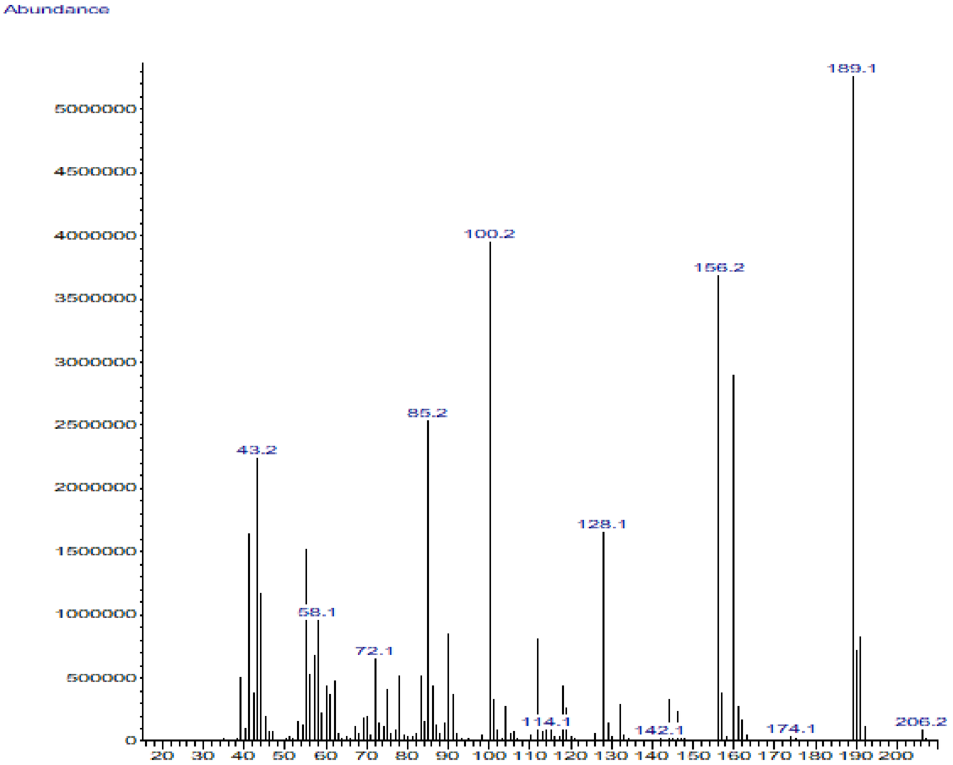

Figure S1. Mass spectrum for the *o*-ethyl hexylcarbamothioate.

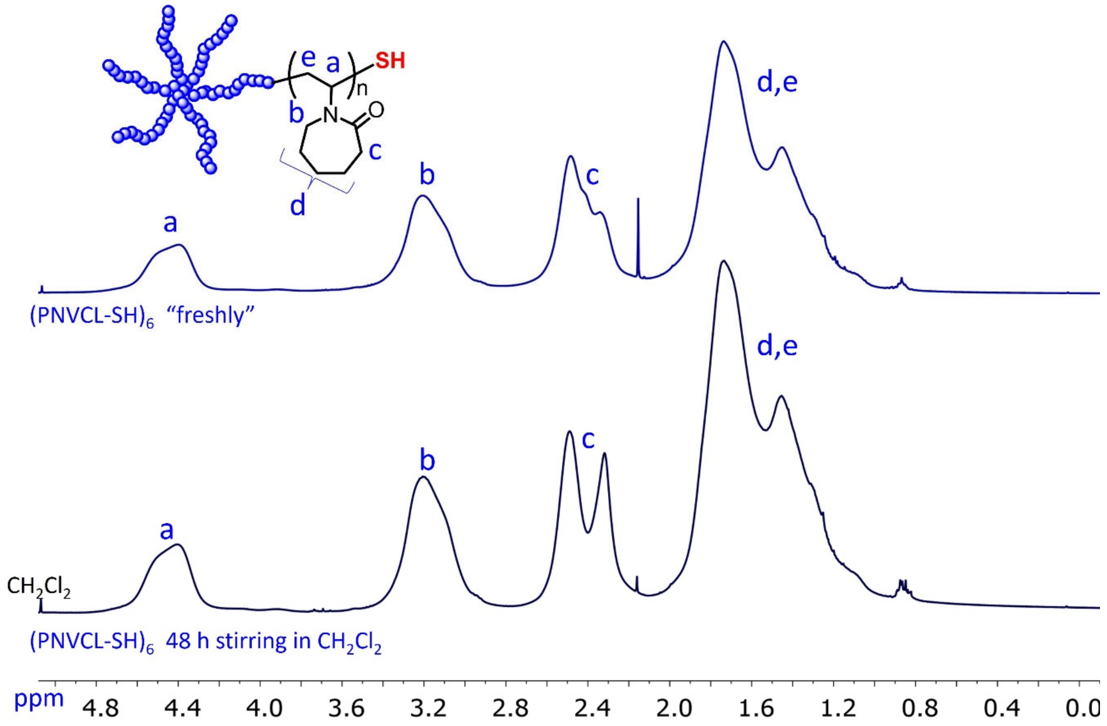

Figure S2. ^{1}H-NMR (400 MHz, $CDCl_3$) spectrum of freshly $(PNVCL)_6$-SH star polymers.

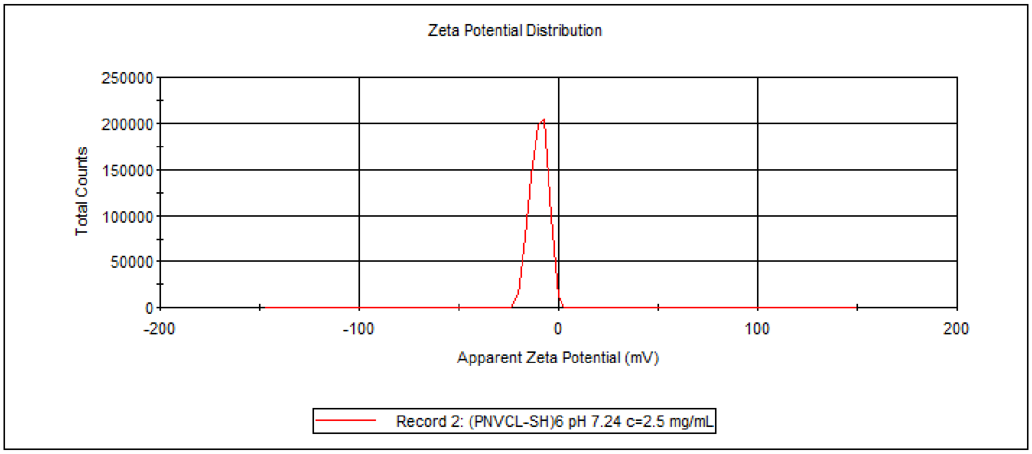

(**a**)

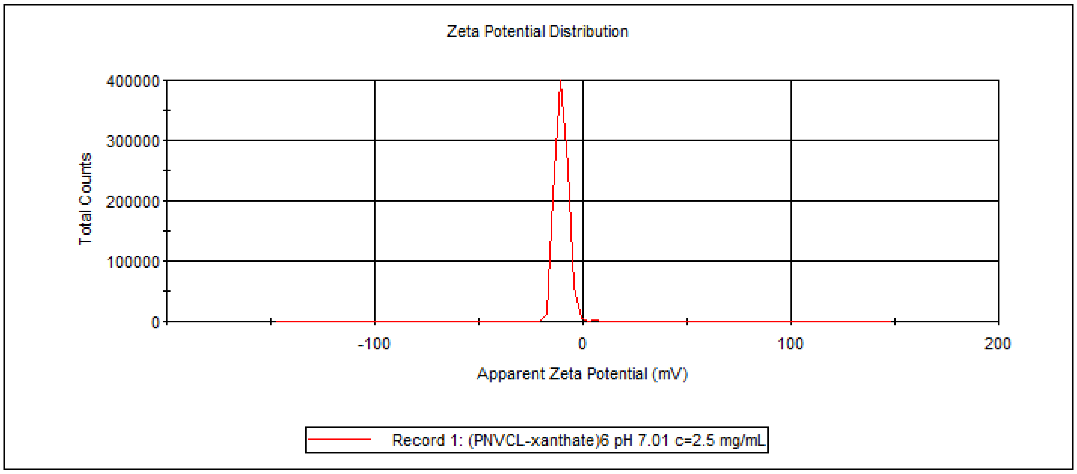

(**b**)

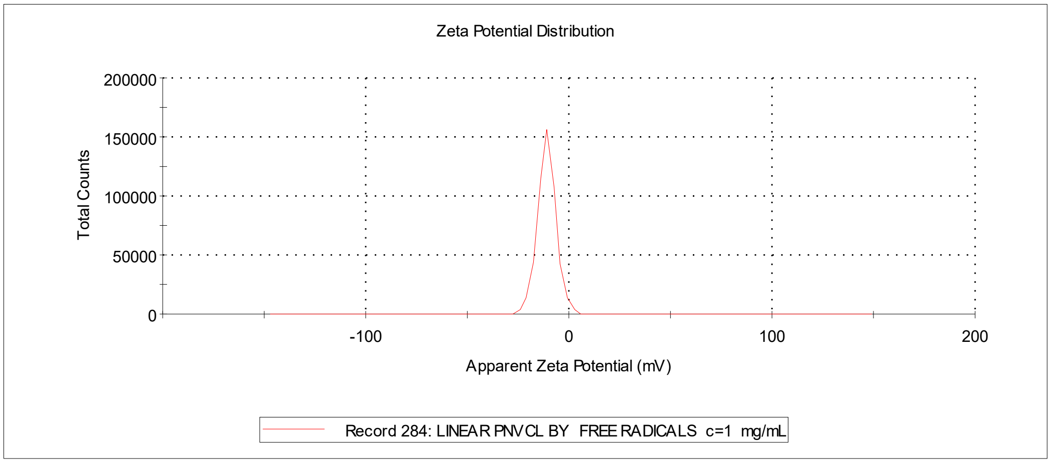

(**c**)

Figure S3. Zeta potential value using DLS for a) $(PNVCL\text{-}SH)_6$ star polymer and b) $(PNVCL_{35}\text{-xanthate})_6$ The samples were dissolved in distilled water (2.5 mg/mL). The pH (unadjusted) of the solution was 7.01 and 7.24 respectively. c) Linear PNVCL polymer prepared by free radical polymerization.
